# An Interdisciplinary Approach for Hypoplastic Amelogenesis Imperfecta: A Case Report

**DOI:** 10.2174/1874210601812010466

**Published:** 2018-06-20

**Authors:** Gulfem Ergun, Ayse Seda Ataol

**Affiliations:** 1 Department of Prosthodontics, Faculty of Dentistry, Gazi University, Çankaya, Ankara, Turkey; 2 Department of Prosthodontics, Faculty of Dentistry, Mersin University, Yenişehir, Mersin, Turkey

**Keywords:** Dental anomalies, Esthetic, Full mouth rehabilitation, Amelogenesis imperfecta, Enamel, Dentine

## Abstract

**Introduction:**

Generally, the appropriate rehabilitation concepts of patients with Amelogenesis Imperfecta (AI) should include a multidisciplinary treatment approach.

**Case Report:**

This case report describes full mouth rehabilitation of a patient with AI. A 20 years old woman was referred to our clinic with a chief complaint of tooth discoloration, diastema, unsatisfactory esthetics and slight tooth sensitivity. Clinical, histological and radiographic examination revealed hypoplastic AI. Short crowns, diastema, occlusal wear with exposed dentin in the posterior areas, the lack of contact points, dental caries and discoloration were the other clinical findings.

**Results:**

As a result of the periodontal and prosthetic evaluation, gingivectomy and ostectomy were planned, and they followed a full mouth fixed prosthetic restoration.

**Conclusion:**

There was no complication or complaint in a 3-year follow-up. At the end of this follow-up period, the patient was satisfied with esthetics, function and phonation properties of her prostheses.

## 1 INTRODUCTION

The dentin and/or enamel can be affected by mineralization defects of dental hard tissues. Amelogenesis Imperfecta (AI) is a rare hereditary disorder which causes structural anomalies in dental enamel [1, 2]. The main characteristics are the loss of tooth structure, especially wear of enamel with exposed dentin at the occlusal aspects of posterior teeth. Lack of enamel tissue can result in various negative clinical situations such as dental sensitivity, tendency for caries formation, attrition and decrease in vertical dimension [3].

The severity of these clinical problems may vary according to the type [4, 5] and the severity of AI [6]. The most frequent type of AI is the hypoplastic type (61.2%) [7] in which all modifications are feasible in combination with different genetic traits. In this form, the radiographic contrast between enamel and dentin is normal [8], and also the thickness of enamel is significantly reduced [8, 9]. Additionally, the longevity of dental restorations in patients with hypoplastic type of AI was higher than the other types [6].

Treatment should begin at childhood and continue into adolescence [5]. Prosthetic, periodontal, orthodontic and surgical treatment concepts could be used according to the type and severity of the patients with AI [2].

The aim of this case report is to describe a full mouth prosthetic rehabilitation concept of a patient with hypoplastic amelogenesis imperfecta. The clinical strategy consisted of a pre-prosthetic diagnosis with Scanning Electron Microscopy (SEM), histological analysis, panoramic radiography and clinical findings. The process was followed by periodontal surgery and prosthetic phase with fixed prosthesis.

## 2 CASE REPORT

**Fig. (1):**
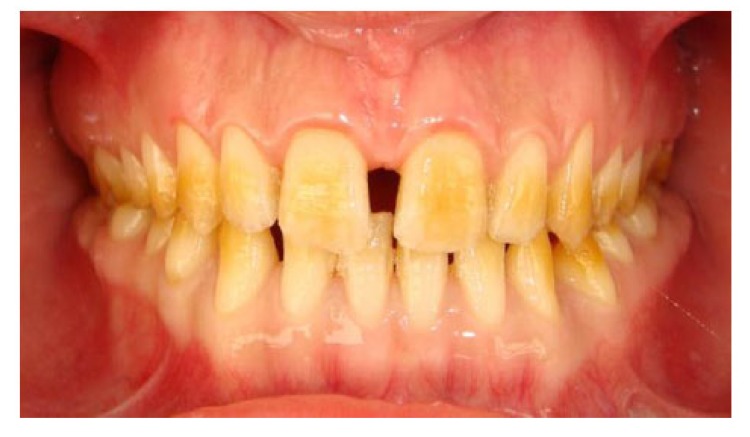
Intraoral frontal view before treatment shows discolored teeth and diastema.

**Fig. (2):**
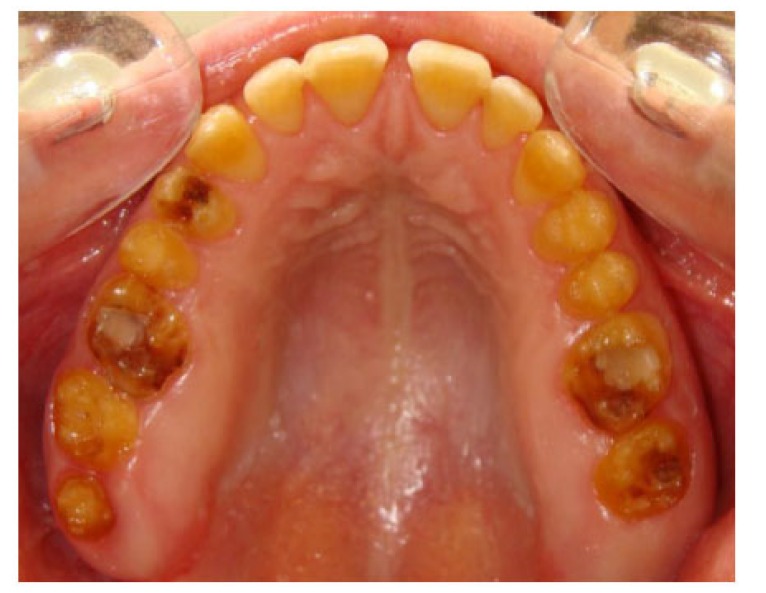
Intraoral occlusal view of upper jaw.

**Fig. (3):**
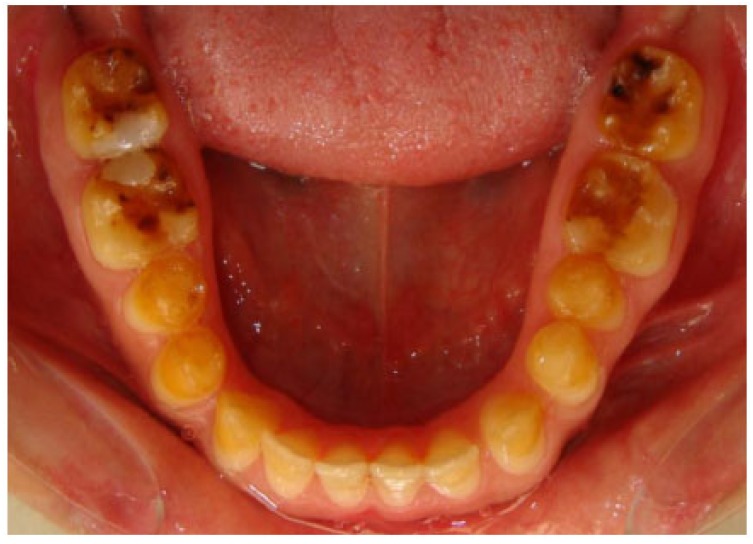
Intraoral occlusal view of lower jaw.

A 20 years old woman was referred to Gazi University Department of Prosthodontics with a chief complaint of tooth discoloration, diastema, unsatisfactory esthetics and slight tooth sensitivity. The medical and dental history revealed that the patient’s family was not affected by AI. A renal ultrasound scan was normal, and it showed no evidence of nephrocalcinosis. Laboratory findings, including serum electrolytes, calcium, phosphate, urea, creatinine, alkaline phosphatase and parathormone levels were all normal. Clinical examination of the patient showed the insufficient enamel thickness, and the patient’s anterior and posterior teeth were discolored (Fig. **1**). The panoramic radiography also showed that the thin enamel layer could not be distinguished from the underlying dentin (Fig. **2**). There were no anterior open bite and missing teeth. However, short crowns, multiple diastema, occlusal wear with exposed dentin in the posterior areas, poor contact points and dental caries are the additional clinical findings (Figs. **2** and **3**). The roots showed normal length and form. The pulp chambers were regular in size. Her oral hygiene was acceptable with no signs of gingivitis (Fig. **1**).

**Fig. (4):**
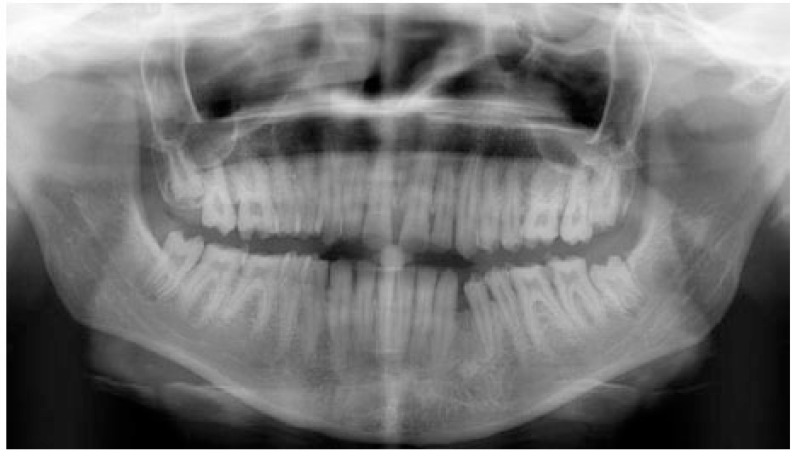
Panoramic radiograph before treatment reveals regular size of pulp chambers, normal length and forms of roots and very thin enamel layer.

**Fig. (5):**
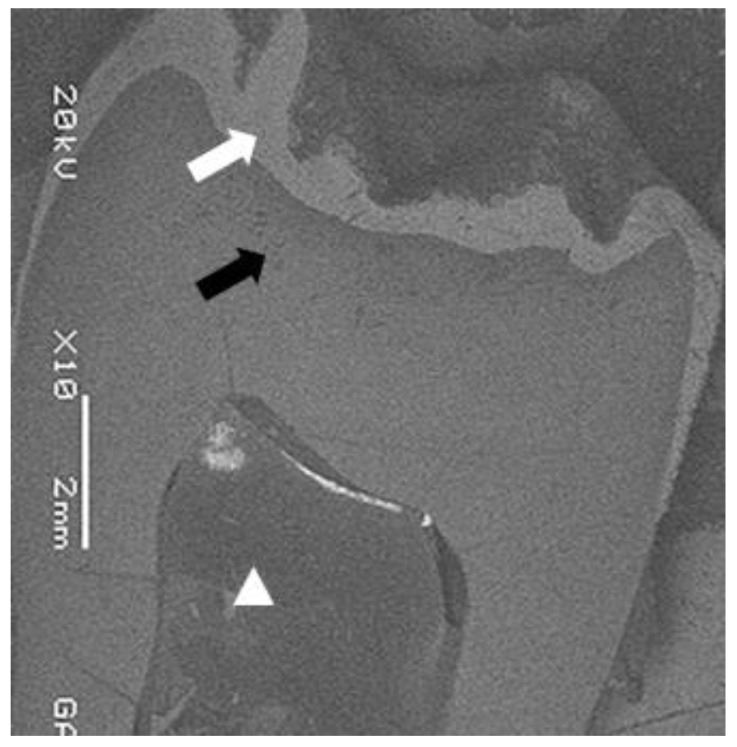
Scanning electron micrograph image of maxillary left third molar shows areas with irregular and thin enamel layer (white arrow), dentin (black arrow) and pulp chamber (white arrowhead).

**Fig. (6):**
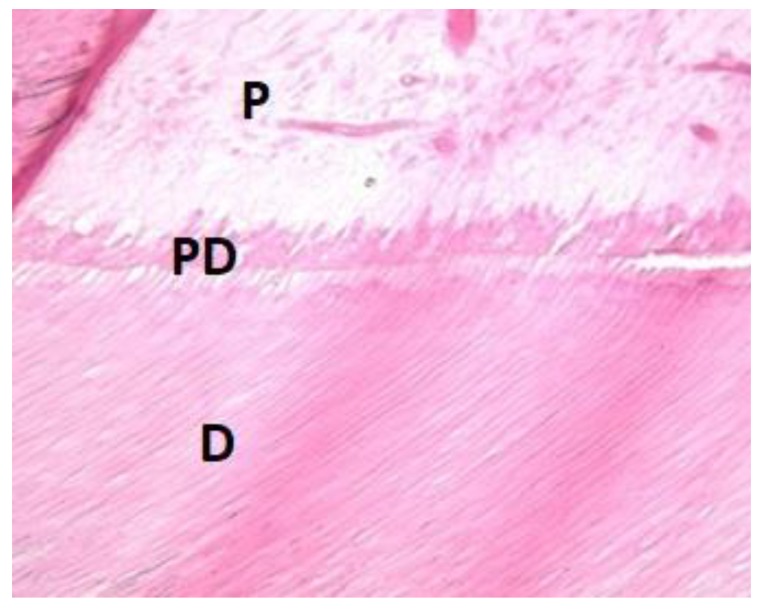
Histologic analysis of mandibular left third molar affected by AI revealed no defect and irregularity in tubular structure of dentin (Hematoxylin-eosin stain) (D=dentin, PD= primer dentin, P=pulp).

The maxillary and mandibular left third molar teeth were extracted to perform SEM and histologic analyzes. These teeth were totally covered by mucosa (Fig. **4**). Therefore, they were selected for SEM and histologic analyzes by the purpose of examining the tooth structure of the patient which had not been exposed to the oral environment. SEM and histologic analyzes were performed on the extracted mandibular and maxillary third molar teeth (Figs. **5** and **6**). One of the third molar teeth was fixed in 4% glutaraldehyde. The tooth was then cut longitudinally, and the sections were coated with gold (Sputter Coater SC7620, Polaron, VG Microtech, England). The analysis was done *via* SEM (JEOL, JSM-6060LV, Tokyo, Japan). SEM analysis showed that there was an insufficient enamel layer (Fig. **3**). Additionally, the other third molar tooth was demineralized with 10% formaldehyde for 3 weeks. The tooth was then cut longitudinally, and it was stained with hematoxylin-eosin stain. Histological findings revealed that dentin structure was intact and there was no irregularity in tubular structure. These findings confirmed that there was no defect related to dentin (Fig. **4**).

**Fig. (7):**
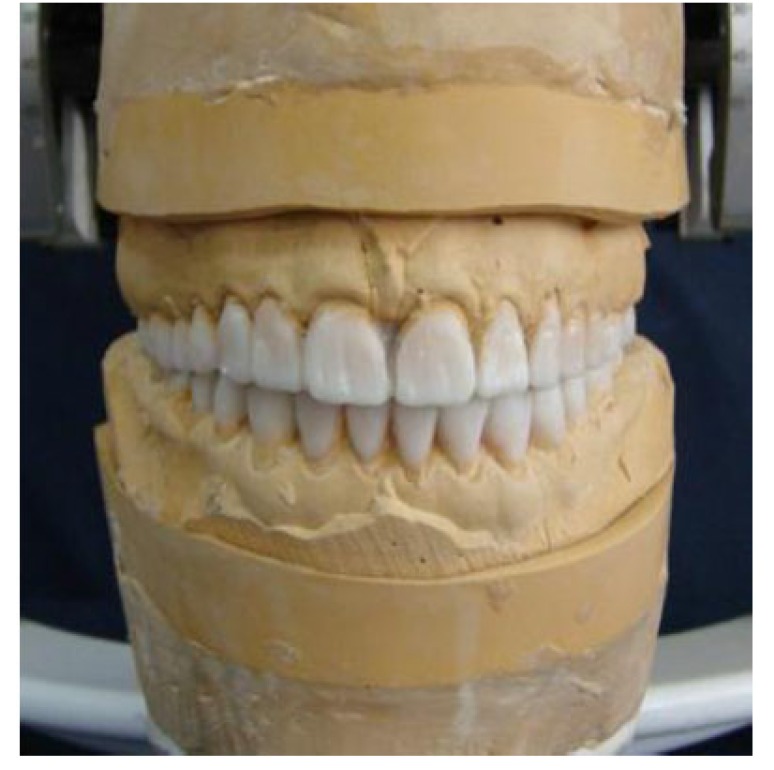
Full mouth diagnostic wax-up of articulated diagnostic models at increased OVD.

Diagnostic casts were attached on a semi-adjustable articulator (Stratos 200; Ivoclar Vivadent Ag, Pforzeim, Germany) in centric occlusion. The occlusal vertical dimension was determined by the Niswonger method [10] and verified with the closest speaking space method. The interocclusal distance at the physiologic rest position was 6 mm. In order to ensure proper vertical dimension and to create enough space for a restorative reconstruction, the bite was opened 5 mm in the anterior region. Furthermore, to predict the last appearance of the restoration, diagnostic wax-up was prepared at the determined vertical dimension (Fig. **7**), and expected treatment outcome was performed with the digital smile design software (Romexis 4.5, Planmeca USA, Inc).

**Fig. (8):**
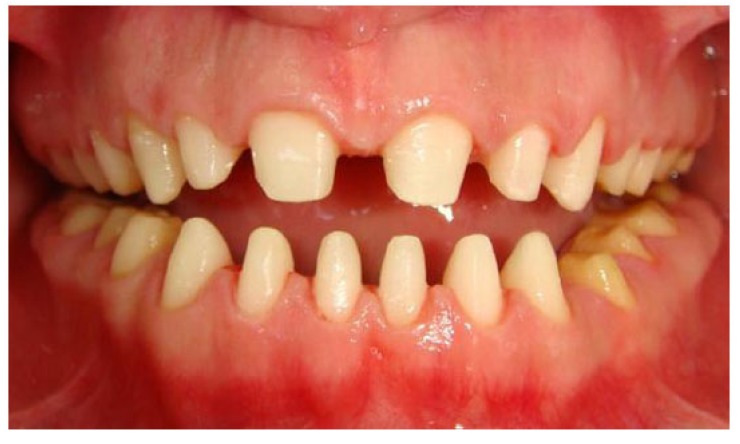
Frontal view of intraoral preparation with chamfer cervical finish line.

**Fig. (9):**
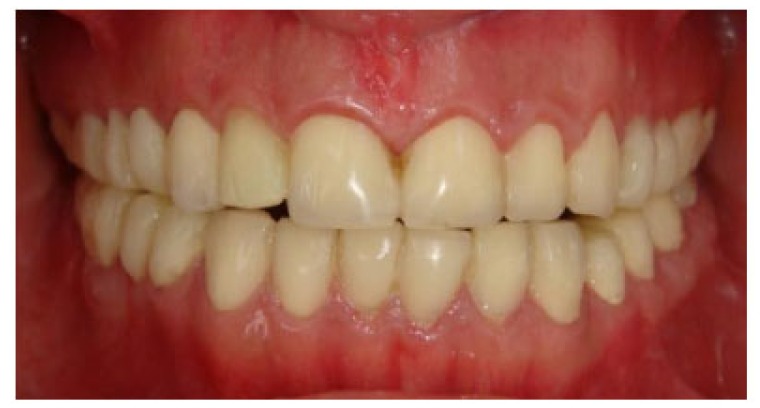
Intraoral view of provisional restorations.

**Fig. (10):**
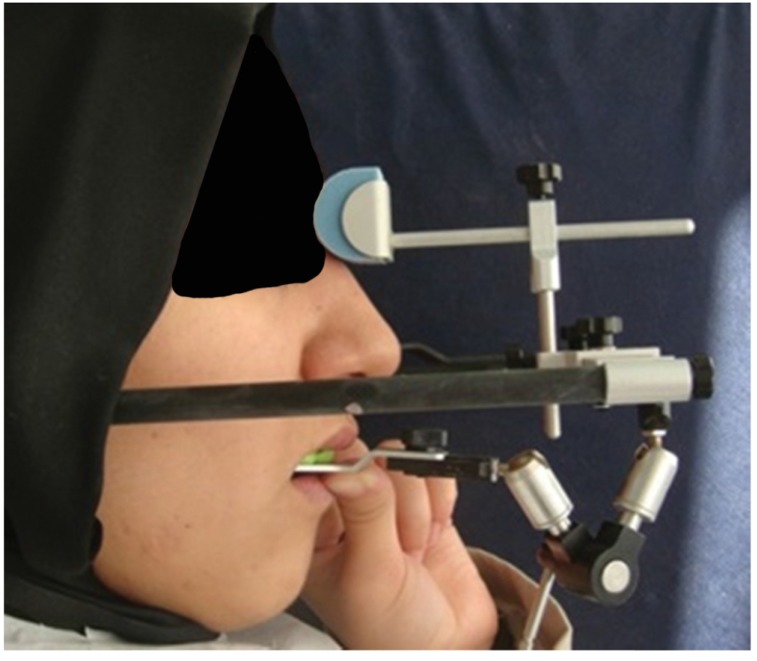
Extraoral view of face bow transfer.

Under local anesthesia, all teeth were prepared with chamfer margins. The crown length of maxillary and mandibular posterior teeth was insufficient for crown retention. In addition, there was an asymmetry of the gingival contours in the anterior maxillary teeth (Fig. **8**). After periodontal evaluation, because of insufficient crown length, ostectomy procedure was performed for maxillary and mandibular posterior teeth. In addition, gingivectomy was applied for maxillary anterior teeth. All periodontal surgery procedures were performed by the periodontologist. The impressions for provisional casts were made with a condensation silicone impression material (Zetaflow; Zhermack, Italy). Then, an interocclusal record was prepared with a hard addition-type A-silicon material (Imprint™ Bite, 3M ESPE) at the increased vertical dimension (5 mm in anterior region) which was determined in diagnostic wax-up stage. The autopolymerizing acrylic resin (ALIKETM; GC America, Alsip, IL, USA) provisional crowns were fabricated at the determined vertical dimension extraorally by an indirect method, then they were cemented with temporary cement (Temp Bond NETM; Kerr) (Fig. **9**). These restorations were assessed in terms of esthetic and phonetics. 1, 2 and 3 month regular checkups were performed. The speech, swallowing, anterior and posterior speaking space, muscle sensitivity, mastication, TMJ discomfort, were assessed during this period. The patient was asymptomatic. The criteria of the success for increased vertical dimension were the absence of pain, no sensitivity in facial and masticatory muscles, phonetic and swallowing satisfaction. At the end of the 3 months follow up period, definitive impressions were made with a condensation silicone impression material (Zetaflow; Zhermack, Italy). Occlusal registration was obtained with a hard addition-type A-silicon material (Imprint™ Bite, 3M ESPE) with a slightly reduced vertical dimension from the provisional restoration. After that, the working casts were obtained and mounted on the semi-adjustable articulator (Stratos 200) using a face-bow transfer (Facebow UTS-3D, Ivoclar Vivadent) (Fig. **10**).

**Fig. (11):**
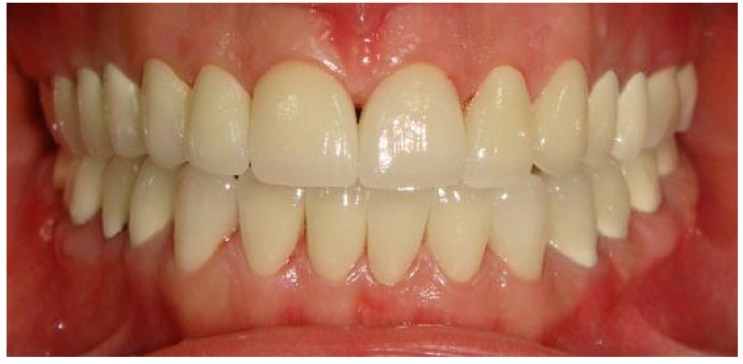
Intraoral view of the patient at the end of the prosthodontic treatment at increased OVD.

The patient was rehabilitated with metal alloy (Mıcrolit Isı, Schütz Dental Group) - ceramic (Cerabien ZR Noritake) fixed partial dentures in the posterior regions. In order to achieve better esthetic appearance and to camouflage the AI affected tooth color, zirconia ceramic (Katana, Kuraray Noritake Dental Inc) based crowns were used in the anterior regions of low and upper jaws (Fig. **11**). With this prosthodontic rehabilitation, maximum interdigitation and a canine guidance could be achieved. Metal-ceramic fixed partial dentures were cemented with zinc polycarboxylate cement (Adhesor® Carbofine; Kerr) and zirconia ceramic crowns cemented with self-etch dual cured resin cement (Maxcem Elite, MXE; Kerr). Following cementation, a maxillary protective occlusal splint was manufactured to protect the restorations from chipping or fracture due to the bruxism. The patient was instructed about oral hygiene maintenance.

## 3 RESULTS

**Fig. (12):**
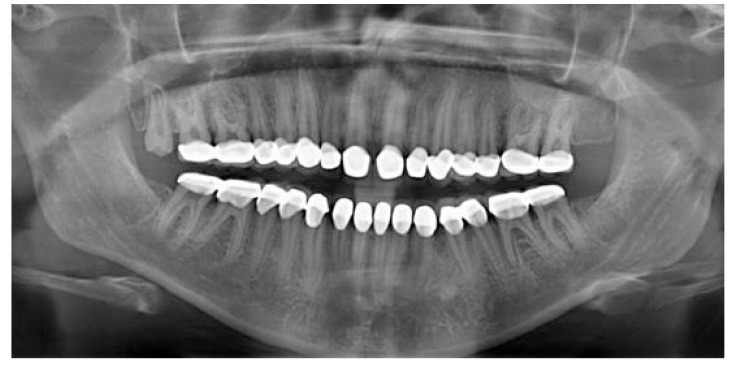
Postoperative panoramic radiograph, zirconia based ceramic crowns in anterior region and metal-ceramic restorations in posterior region.

**Fig. (13):**
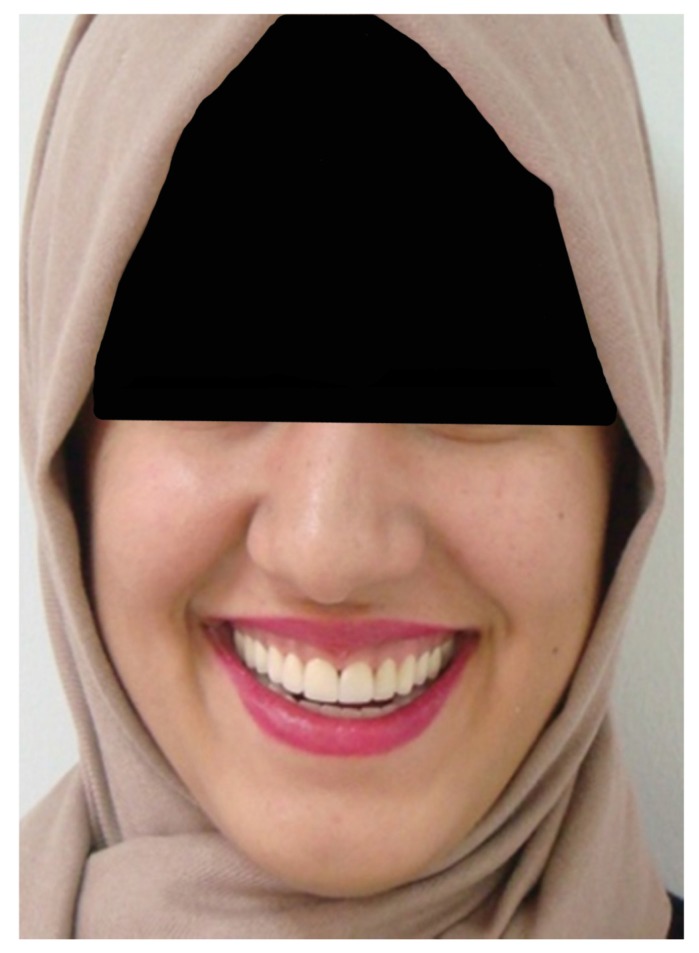
Final facial photograph taken after treatment. The patient was very satisfied with the results in terms of both esthetic, function and phonation.

After full mouth prosthetic rehabilitation, mastication capacity and facial appearance were improved, and there was no tooth sensitivity or complication related to temporomandibular joint. Radiographical and clinical examinations revealed no evidence of disorders associated with the restored teeth or periodontal structures (Figs. **12** and **13**). The regular clinical and radiographic follow up have been carried out at 3^rd^, 6^st^, 12^nd^, 24^th^ months and 3^rd^ year postoperatively with visual and radiographic examinations. A 3-years follow-up revealed that the patient was still pleased with function, dental esthetics and facial harmony.

## 4 DISCUSSION

The patients suffering from AI have a great number of clinical problems that affect their life quality. The rehabilitation of the patients with AI is a challenge which requires a multidisciplinary approach [2].

The evaluation of extracted third molar teeth with histological and SEM evaluation has revealed the diagnosis of AI. Histological evaluation showed that the dentin structure was intact (Fig. **6**). Although regional enamel loss was observed clinically, SEM analysis showed that there was insufficient enamel layer with irregular structure, and there was no interruption in the enamel layer (Fig. **5**). The difference between intraoral condition and the results of SEM analysis was considered to be related to the fact that the third molar tooth used for SEM analysis was impacted.

When the skeletal growth is completed, it is reported that full-mouth prosthetic rehabilitation with all-ceramic or porcelain-fused to metal restorations seems to be the best treatment option for AI. Due to the fact that soft and hard tissue are completed until 18-20 years old [11], full mouth fixed prosthetic restoration has been planned to restore the dentition.

In the present case, there was a need to increase vertical dimension to provide an adequate interocclusal space. The patient can adapt to an increase of vertical dimension up to 5 mm [12]; however, it is impossible to specify the upper limit. Moreover, limiting the increase in vertical dimension is important to avoid the complexity of the prosthodontic treatment [13]. In the current case report, Occlusal Vertical Dimension (OVD) increased 5 mm in the anterior region, and 3 to 4 mm in the posterior region respectively.

The crown height of the maxillary and mandibular posterior teeth needed to be lengthened to increase surface area for retention of restorations [14]. The crown lengthening procedure allows developing a proper form for a restoration. In addition, the patient’s smile can be enhanced by manipulating the gingival contour. This procedure involves either a gingivectomy or an ostectomy to obtain biologic width for restoration and crown placement. The choice between gingivectomy or ostectomy depends on some factors such as the width of attached gingiva [14, 15]. In the present case report, ostectomy was applied to maxillary and mandibular posterior teeth due to the insufficient width of attached gingiva and to obtain biological width. In addition, gingivectomy was applied to the maxillary anterior teeth with the aim of providing gingival symmetry. In the present case report, surgical crown lengthening and gingivectomy were preferred.

The time of using a provisional prosthesis varied from 2 to 6 months in the previous studies [13, 16]. In addition, Hempton and Dominici [15] reported that the free gingival margin requires minimum 3 months to establish its final vertical position. In the present report, the provisional restorations were used for 3 months, after the periodontal surgery, to restore the lost OVD, to evaluate the adaptation, the temporomandibular discomfort of the patient and to establish the final contour of gingiva.

The patient was rehabilitated with a full-mouth fixed partial denture, zirconia based ceramic crowns in the anterior region and metal-ceramic restorations in the posterior region (Fig. **11**). Zirconium oxide-based restorative materials have improved mechanical properties and low bacterial adhesion. In addition, to camouflage the underlying discoloration of tooth structure and to restore anterior region, zirconia based all ceramic crowns were preferred for improved esthetics. Although studies support the use of zirconia based all ceramic restorations for posterior regions [17, 18], the most common complications were veneering ceramic fractures for zirconia based all ceramic restorations [19]. Alternative restoration techniques like monolithic zirconia restorations are options to eliminate the increased risk of veneering ceramic fractures. Monolithic zirconia ceramic restorations are being used in high load bearing areas [20]. Although monolithic zirconia restorations offer above mentioned advantages, metal-ceramic restorations were used in the posterior areas due to the high cost of monolithic zirconia restoration and the difficulty on the repair of fracture type. There was a slight midline deviation. However, the patient did not accept orthodontic treatment.

In this case, new centric occlusion was adjusted according to the patient’s centric relation since it is the only reproducible position and posterior teeth can be loaded axially in centric occlusion [9]. The occlusion was constructed as mutually protected occlusion without eccentric contacts to prevent the restorations during functional and parafunctional movements.

After full mouth prosthetic rehabilitation, mastication capacity and facial appearance were improved. The patient was encouraged to attend regular recall appointments because of applying a comprehensive restoration and the necessity to follow the young adult patient during the adaptation period. The regular clinical and radiographic follow up have been carried out at the 3^rd^, 6^st^, 12^nd^, 24^th^ months and 3^rd^ years.

## CONCLUSION

Management of amelogenesis imperfecta using fixed prosthodontics is reasonable for restoring the function and esthetic appearance. The long-term efficiency of fixed prosthodontics has advantages to prevent further destructions related to occlusal wear or impairment of the vertical growth.
